# Protein coalitions in a core mammalian biochemical network linked by rapidly evolving proteins

**DOI:** 10.1186/1471-2148-11-142

**Published:** 2011-05-25

**Authors:** Chrysanthi Ainali, Michelle Simon, Shiri Freilich, Octavio Espinosa, Lee Hazelwood, Sophia Tsoka, Christos A Ouzounis, John M Hancock

**Affiliations:** 1 Centre for Bioinformatics, Department of Informatics, School of Natural and Mathematical Sciences, King's College London, Strand, London WC2R 2LS, UK; 2 Bioinformatics Group, MRC Harwell, Harwell Science and Innovation Campus, Oxfordshire OX11 0RD, UK; 3 The Blavatnik School of Computer Sciences & Sackler School of Medicine, Tel Aviv University, Ramat Aviv 69978, Israel; 4 Department of Biochemistry, University of Oxford, UK, South Parks Road, Oxford, OX1 3QU, UK; 5 Institute of Cancer Research, Chester Beattie Laboratories, London SW3 6JB, UK; 6 Computational Genomics Unit, Institute of Agrobiotechnology, Centre for Research & Technology Hellas (CERTH), GR-57001 Thessalonica, Greece

## Abstract

**Background:**

Cellular ATP levels are generated by glucose-stimulated mitochondrial metabolism and determine metabolic responses, such as glucose-stimulated insulin secretion (GSIS) from the β-cells of pancreatic islets. We describe an analysis of the evolutionary processes affecting the core enzymes involved in glucose-stimulated insulin secretion in mammals. The proteins involved in this system belong to ancient enzymatic pathways: glycolysis, the TCA cycle and oxidative phosphorylation.

**Results:**

We identify two sets of proteins, or protein coalitions, in this group of 77 enzymes with distinct evolutionary patterns. Members of the glycolysis, TCA cycle, metabolite transport, pyruvate and NADH shuttles have low rates of protein sequence evolution, as inferred from a human-mouse comparison, and relatively high rates of evolutionary gene duplication. Respiratory chain and glutathione pathway proteins evolve faster, exhibiting lower rates of gene duplication. A small number of proteins in the system evolve significantly faster than co-pathway members and may serve as rapidly evolving adapters, linking groups of co-evolving genes.

**Conclusions:**

Our results provide insights into the evolution of the involved proteins. We find evidence for two coalitions of proteins and the role of co-adaptation in protein evolution is identified and could be used in future research within a functional context.

## Background

Eukaryotic organisms make use of complex biochemical pathways to maintain homeostatic processes in response to changes in their environment. These responses can be broadly divided into gene regulatory responses, whereby changes in an environmental condition give rise to changes in gene expression, and metabolic responses, which result from the responses of metabolic networks to environmental changes. Many metabolic adaptations to environmental changes are mediated by genetic regulation.

Pancreatic β-cells are the cells responsible for insulin secretion in vertebrates and therefore play a key role in glucose homeostasis. It has been suggested that glucose homeostasis evolved to protect brains from hypo- and hyperglycaemic effects [1]. However, Diptera secrete insulin-like proteins and there is evidence for conservation of insulin signalling pathways [2] and a role in regulating glucose and trehalose levels in the haemolymph [3,4], suggesting that the insulin secretion and signalling pathways pre-date the origin of chordates and their complex, glucose-dependent brain physiology. The evolution of the pancreas and its β-cells is well studied and has been reviewed elsewhere [5]. Hagfish and lampreys have a pancreas made up almost entirely of β-cells and the cell type itself is present in Amphioxus, a Cephalochordate, where it is associated with the intestinal tissue in a dispersed manner.

Glucose-stimulated insulin secretion (GSIS) from the β-cells of pancreatic islets is a metabolic response that is critically dependent on cellular ATP levels generated by glucose-stimulated mitochondrial metabolism. In pancreatic β-cells, ATP production is proportional to glucose uptake by the cell, which in turn is proportional to the glucose concentration in the surrounding body fluids [6]. A complex network of reactions in both the cytoplasm and the mitochondrion modulates ATP production. We recently described a kinetic model of the biochemical processes leading from glucose uptake to ATP production in this cell type [7]. Many of the components in this biochemical network are ancient enzymes with evolutionary origins in bacteria and therefore predate the invention of pancreatic β-cells. Here we consider the evolution of the components of this system as an example of an element of core cellular biochemistry that is also used for a cell-type-specific function.

There have been evolutionary studies of certain individual components of the GSIS system. Phylogenetic analysis of metabolic evolution [8,9] suggests that the TCA cycle may have evolved in at least two steps: an initial phase, in which the synthesis of oxaloacetate from oxoglutarate occurred but in which the cycle was not closed, followed by closure of the cycle after the accumulation of atmospheric oxygen. Linkage of glycolysis to the TCA cycle took place at approximately the same time as completion of the cycle itself. It has been suggested that many bacterial genomes lack a complete TCA cycle, which in turn might suggest the use of alternative pathways in different lineages [10]. Glycolysis has been well studied, most recently in the context of whole genome duplications [11]. In that context, duplications were identified at the root of the vertebrate tree for most of the glycolytic enzymes. The exceptions to this were triosephosphate isomerase and phosphoglucose isomerase, which only showed evidence of duplication in fish [11]. Another study has considered the evolution of 78 nuclearly encoded oxidative phosphorylation genes and demonstrated that gene duplicates were relatively rare in these gene families [12]. Despite these studies, no integrated study of this system has been carried out.

Numerous forces can act on the evolution of metabolic pathways such as the GSIS network, including pressures affecting evolutionary rate in a systematic manner, gene duplication, positive and purifying selection, or gene expression [13]. Additionally, genes can be categorized by phylogenetic age, corresponding to the evolutionary distance over which homologues can be detected [14]. Here, we consider the effects of these various influences on the recent evolution of the entire GSIS system, allowing us to consider differences and similarities between its component parts. We find evidence for two coalitions of proteins, corresponding to groups of biochemical pathways, which appear to have coevolved both in divergence rate and duplication history. We also identify some rapidly evolving proteins which appear to form the interfaces between the detected protein coalitions, perhaps acting as evolutionary "adapters".

## Results

The 48 reactions identified in the GSIS model of core pancreatic β-cell biochemistry are catalysed by a minimum of 77 protein components based on current biochemical knowledge [7]. This biochemical network includes a number of large macromolecular complexes including the cytochrome c oxidase complex (complex IV; 10 components) and the NADH:ubiquinone oxidoreductase complex (complex I; 9 components). On the other hand, for some of the reactions in the network we were unable to identify corresponding proteins. Most of these unidentified proteins are involved in metabolite transport across the mitochondrial membrane and are likely to be unidentified membrane proteins. In total, we were able to identify 69 GSIS protein components whose evolution we could study.

### Antiquity of GSIS core genes

**Figure 1:**
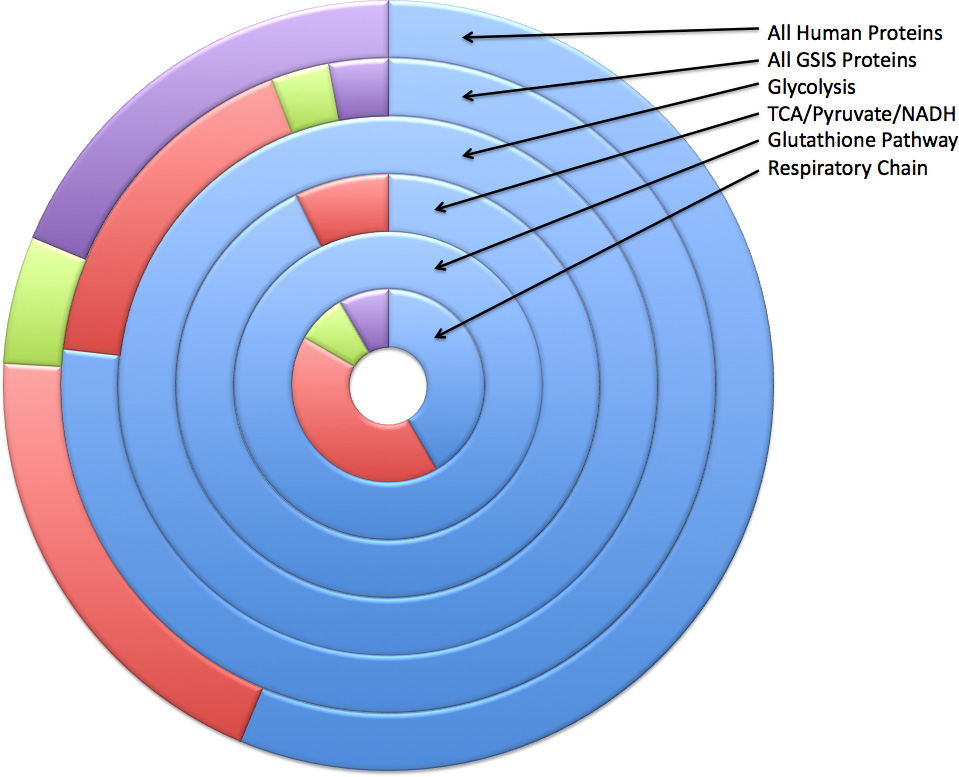
**Phylogenetic classification of GSIS genes**. The proportions of genes in individual sub-pathways of the GSIS model are compared with the proportions seen for all human proteins. Blue = Universal; Red = Eukaryotic; Green = Metazoan; Purple = Vertebrate.

To characterise the evolutionary dynamics of the GSIS system, we first considered the antiquity of the individual genes that comprise it. Antiquity is defined as the taxonomic distance over which homologues of the mammalian genes could be detected searching the COGENT database [15]. Using this process, we assigned each enzyme gene to one of four phylogenetic groups: Universal (U), Eukaryote-specific (E), Metazoan-specific (M) and Vertebrate-specific (V) [16]. Of the 69 enzyme genes assigned to phylogenetic categories (Figure 1), 77% were classified as Universal, 17% as Eukaryotic, 3% as Metazoan and 3% as Vertebrate. Comparison of the relative proportions of U, E, M and V genes in the GSIS network to the whole metabolic complement of the human genome (56%, 20%, 5%, 19% respectively - [17], Table 1) indicates that the GSIS system contains a higher proportion of U genes and fewer V genes. This difference is statistically significant, with a P-value < 0.002 (chi-squared, df = 3). When the assignment of proteins to pathways is considered, there appear to be clear differences for different pathways. Most pathways are predominantly Universal. However the respiratory chain contains a mix of genes of different phylogenetic categories and relatively few Universal ones (Figure 1).

**Table 1:** Proteins considered in this analysis

Protein (Abbreviation)	PID (Hs)^1^	Path-way^2^	Class^3^	H-M^4^
Glucokinase (GK)	P35557	G	U	98.3
6-phosphofructokinase (F6P)	P17858	G	U	97.0
fructose-bisphosphate aldolase (A) (FBa)	P04075	G	U	98.9
fructose-bisphosphate aldolase (B) (FBb)	P05062	G	U	99.7
fructose-bisphosphate aldolase (C) (FBc)	P09972	G	U	98.6
glyceraldehyde 3-phosphate dehydrogenase (GAPD)	P04406	G	U	97.0
bisphosphoglycerate phosphatase (1) (PGP1)	P18669	G	U	99.6
bisphosphoglycerate phosphatase (2) (PGP2)	P15259	G	U	95.2
Pyruvate kinase (PK)	P30613	G	U	95.5
Lactate dehydrogenase A (LDHa)	P00338	G	U	97.6
Lactate dehydrogenase B (LDHb)	P07195	G	U	99.1
Pyruvate Dehydrogenase Complex A (PDCa)	P08559	T	U	99.5
Pyruvate Dehydrogenase Complex B (PDCb)	P11177	T	U	97.5
Pyruvate Dehydrogenase Complex C (PDCc)	P29803	T	U	87.2
Citrate Synthase (CS)	O75390	T, P	U	97.4
Aconitase (ACO)	Q99798	T, P	U	98.8
Isocitrate Dehydrogenase (NAD+) (A) (IDHa)	P50213	T, P	U	98.6
Isocitrate Dehydrogenase (NAD+) (G) (IDHg)	P51553	T, P	U	97.2
Oxoglutarate Dehydrogenase Complex (OGDC)	Q02218	T	U	94.1
Succinyl-CoA synthetase A (SCSa)	P53597	T	U	97.0
Succinyl-CoA synthetase B (SCSb)	Q96I99	T	U	96.1
Succinate Dehydrogenase A (SDHa)	P31040	T	U	94.0
Succinate Dehydrogenase B (SDHb)	P21912	T	U	97.0
Fumarase (FM)	P07954	T, N	U	95.3
Malate Dehydrogenase (mitochondrion & cytosol) (MDH)	P40926	T, N	U	97.6
Alanine Transaminase (AlaTA)	P24298	N	U	92.5
Aspartate Transaminase (AspTA)	P00505	N	U	98.4
Nucleoside Diphosphate Kinase (NDK)	O00746	T	U	87.8
NADH: Ubiquinone oxidoreductase 1 (MT-ND1)	O15239	R	E	92.9
NADH: Ubiquinone oxidoreductase 2 (MT-ND2)	O43678	R	E	93.9
NADH: Ubiquinone oxidoreductase 3 (MT-ND3)	O95167	R	E	88.1
NADH: Ubiquinone oxidoreductase 4 (MT-ND4)	O00483	R	E	92.7
NADH: Ubiquinone oxidoreductase 5 (MT-ND5)	Q16718	R	U	91.3
NADH: Ubiquinone oxidoreductase 6 (MT-ND6)	P56556	R	U	90.8
NADH: Ubiquinone oxidoreductase 7 (MT-ND7)	O95182	R	E	95.5
NADH: Ubiquinone oxidoreductase 8 (MT-ND8)	P51970	R	U	94.7
NADH: Ubiquinone oxidoreductase 9 (MT-ND9)	Q16795	R	U	87.3
Ubiquinol: Cytochrome c Oxidoreductase H (UQCRH)	P08574	R	U	92.3
Ubiquinol: Cytochrome c Oxidoreductase I (UQCRI)	P47985	R	U	94.5
Ubiquinol: Cytochrome c Oxidoreductase Q (UQCRQ)	O14949	R	E	85.2
Ubiquinol: Cytochrome c Oxidoreductase R1 (UQCRR1)	O14957	R	M	92.7
Ubiquinol: Cytochrome c Oxidoreductase C2 (UQCRC2)	P22695	R	U	93.2
Cytochrome c Oxidase 6B1 (Cox6B1)	P14854	R	E	96.5
Cytochrome c Oxidase 2* (Cox2)	P00403	R	U	86.8
Cytochrome c Oxidase 8a (Cox8a)	P10176	R	V	85.5
Cytochrome c Oxidase 5A (Cox5a)	P20674	R	U	88.0
Cytochrome c Oxidase 4A (Cox4a)	P13073	R	E	89.9
Cytochrome c Oxidase 6C (Cox6c)	P09669	R	M	86.7
Cytochrome c Oxidase 7B (Cox7b)	P24311	R	V	88.8
Cytochrome c Oxidase 1* (Cox1)	P00395	R	U	95.5
Cytochrome c Oxidase 7R (Cox7r)	O14548	R	E	93.0
Cytochrome c Oxidase 7C (Cox7c)	P15954	R	E	88.9
Oxoglutarate Carrier (OGC)	Q02978	N, P	E	97.8
Citrate Carrier (CIC)	P53007	M	E	96.5
Nicotinamide nucleotide transhydrogenase (NNT)	Q13423	S	U	96.2
Glutathione reductase (GSSGR)	P00390	S	U	89.3
Glutathione peroxidase (GSSGP)	P07203	S	U	90.6
Glycerol-3-phosphate dehydrogenase (FAD dependent) (GUT2P)	P43304	N	U	97.0
Glycerol-3-phosphate dehydrogenase (NAD+) (G3PD)	P21695	N	U	96.8
Malate Dehydrogenase (oxaloacetate-decarboxylating) (NADP+) X (MEx)	P48163	P	U	94.9
Malate Dehydrogenase (oxaloacetate-decarboxylating)(NADP+) N (Men)	Q16798	P	U	96.4
ATP/ADP Carrier 2 (AAC)	P05141	M	U	99.3
cytosolic Isocitrate Dehydrogenase (NADP+) P (IDHcp)	P48735	P	U	82.0
cytosolic Isocitrate Dehydrogenase (NADP+) C (IDHcc)	O75874	P	U	97.8
Pyruvate Carboxylase (PC)	P11498	T, P	U	98.6
ETF:Q oxidoreductase (ETF-QO)	Q16134	N	U	95.8
Manganese-dependent superoxide dismutase 2 C (SOD2c)	P00441	S	U	88.9
Manganese-dependent superoxide dismutase 2 M (SOD2 m)	P04179	S	U	91.9
Manganese-dependent superoxide dismutase 2 E (SOD2e)	P08294	S	U	75.2

^1 ^Uniprot Accession ID for the human protein

^2 ^Pathway designation: G = glycolysis, T = TCA cycle, R = respiratory chain, N = NADH shuttle, P = pyruvate cycle, M = metabolite transport, S = glutathione pathway

^3 ^Phylogenetic class

^4 ^Human-mouse sequence similarity (%)

The construction of phylogenetic profiles also allowed the identification of human proteins that share homologues in other genomes, indicating a history of ancient gene duplication. This analysis identified six protein clusters. The largest of these comprised the four isocitrate dehydrogenases (IDHa, IDHg, IDHcp, IDHcc), which shared 31 homologues in other genomes. Other groups also consisted of functionally related proteins: pyruvate dehydrogenase complex subunits (PDCc & PDCa; 207 shared homologues), other dehydrogenases (MDH, LDHa, LDHb; 89 shared homologues), and superoxide dismutases (SOD2 m & SOD2e; 80 shared homologues). Two weaker linkages were also detected between less obviously related proteins: ETF:Q oxidoreductase (ETF-QO) and glutathione reductase (GSSGR) (7 shared homologues) and pyruvate dehydrogenase subunit B (PDCb) and cytochrome c oxidase subunit 5A (Cox5A) (2 shared homologues). Searches of the Pfam database [18] identified common domain compositions underlying most of these relationships. IDHs all contain the Iso_dh domain (PF00180), PDCs (a and c) contain the E1_dh domain (PF00676), MDH and LDHs share two domains, Ldh_1_N (PF00056) and Ldh_1_C (PF02866) and the SODs share the Sod_Cu domain (PF00080). Structural relationships between the more weakly linked proteins were less clear. ETF-QO and GSSGR both contain domains classified as part of the FAD/NAD(P)-binding Rossmann fold superfamily (CL0063) but no shared domains were identified between PDCb and Cox5A, most likely indicating a false positive observation.

### Sequence conservation

The preceding analysis indicates that a high proportion of GSIS proteins have been conserved over long periods of evolutionary time. We therefore investigated whether this was reflected in their level of sequence conservation. We did this by measuring conservation between human and mouse orthologues, as a proxy for the overall level of evolutionary conservation. We used this pair of species because of the quality of their genome sequences and because their divergence time (c. 90 MYA) is short enough that they will show relatively little mutational saturation. Of 77 enzyme genes for which we searched for human and mouse orthologues, we could identify 69 orthologue pairs in the two species. As mentioned, the missing genes primarily reflect absence of knowledge of the identity of some membrane transporters in the system (see also Materials & Methods).

**Figure 2:**
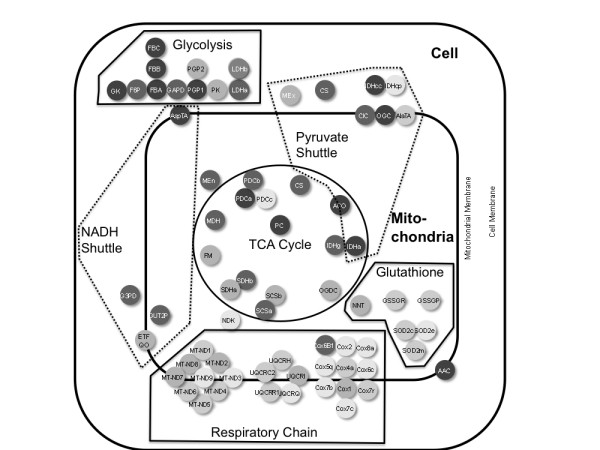
**Schematic representation of protein conservation in the GSIS biochemical network**. Proteins are represented as circles with shading representing their level of sequence similarity between human and mouse (see Table 1). Darkly shaded proteins are most similar and lightly shaded proteins least similar. Five shades of grey are used to represent the five quintiles of the sequence similarity distribution. Proteins are separated between cytoplasm, mitochondrial membrane and mitochondrial matrix, grouped together into sub-pathways and co-localised to give an approximate representation of their functional relationships.

We determined sequence similarity between these orthologs at the protein level, as this is the most relevant measure for identifying functional conservation. Individual values for protein sequence similarity for each enzyme are presented in Table 1 and summarised graphically in Figure 2. Details of the GSIS biochemical network are given in [7] and the Additional File 1. The mean human-mouse conservation across these proteins was 93.7% (standard deviation 4.9%), which is considerably greater than the widely accepted average of 85% [19], but there was significant variability - the lowest conservation observed was 75.2% for Manganese-dependent superoxide dismutase 2E, while five proteins showed conservation of > 99%. Careful examination of the alignments of the less conserved proteins confirmed that the low levels of conservation observed in these proteins were not alignment artifacts. Three of the five very highly conserved proteins, bisphosphoglycerate phosphatase (1), fructose-bisphosphate aldolase (A) and lactate dehydrogenase B, are components of the cytoplasmic glycolysis pathway, which computational analysis suggests plays a critical role in controlling the activity of the GSIS network [7]. The other two, ATP/ADP carrier 2 and Pyruvate Dehydrogenase Complex A, are proteins of the metabolite transport and TCA cycle pathways, which take place in mitochondria. The two mitochondrially encoded oxidative phosphorylation genes analysed here (Cox 1 & Cox 2) did not show lower conservation than the other genes considered (mean 91.15%), consistent with previous observations [20].

**Figure 3:**
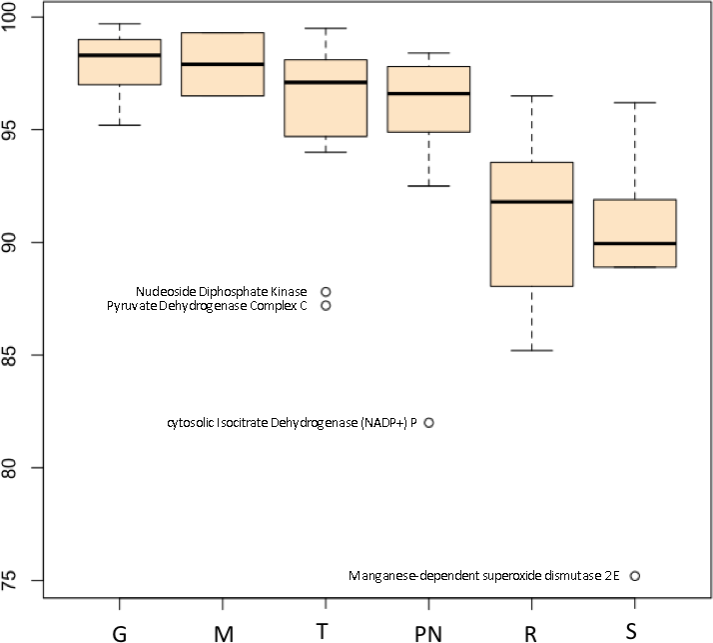
**Effect of sub-pathway membership on sequence divergence of GSIS proteins**. Tukey's boxplot of the results of ANOVA analysis on the effect of sub-pathway membership on sequence divergence of GSIS proteins. G = glycolysis, M = metabolite transport, R = respiratory chain, S = glutathione pathway, TPN = TCA cycle+pyruvate cycle+NADH shuttle.

To further characterise the distribution of conservation across the identifiable sub-pathways and phylogenetic categories in the model we carried out an analysis of variance (ANOVA). Of five treatment variables (pathway membership, phylogenetic classification, the number of gene duplications observed in the Ensembl gene trees, the presence/absence of positive selection in these trees, and known association or otherwise with human disease), only sub-pathway membership had a significant effect on divergence (F = 7.82; df = 5; P < 0.001). A Tukey HSD test showed significantly lower sequence conservation (adjusted P < 0.05) for glutathione pathway and respiratory chain proteins than for members of the glycolysis and TCA cycle groups. The glutathione pathway proteins also showed significantly lower conservation than the pyruvate and NADH shuttle proteins. Taken together, these results suggest that there are two clusters of conservation level in the data set - on the one hand glycolysis, metabolite transport, TCA cycle and pyruvate/NADH shuttles and on the other hand the respiratory chain and the glutathione pathway. This distinction is illustrated in the Tukey's boxplot (Figure 3), which shows the glutathione and respiratory chain pathways with lower and more variable conservation values than the other sub-pathways. The distinction is also evident in Figure 2.

### Gene Duplication events and positive selection

**Figure 4:**
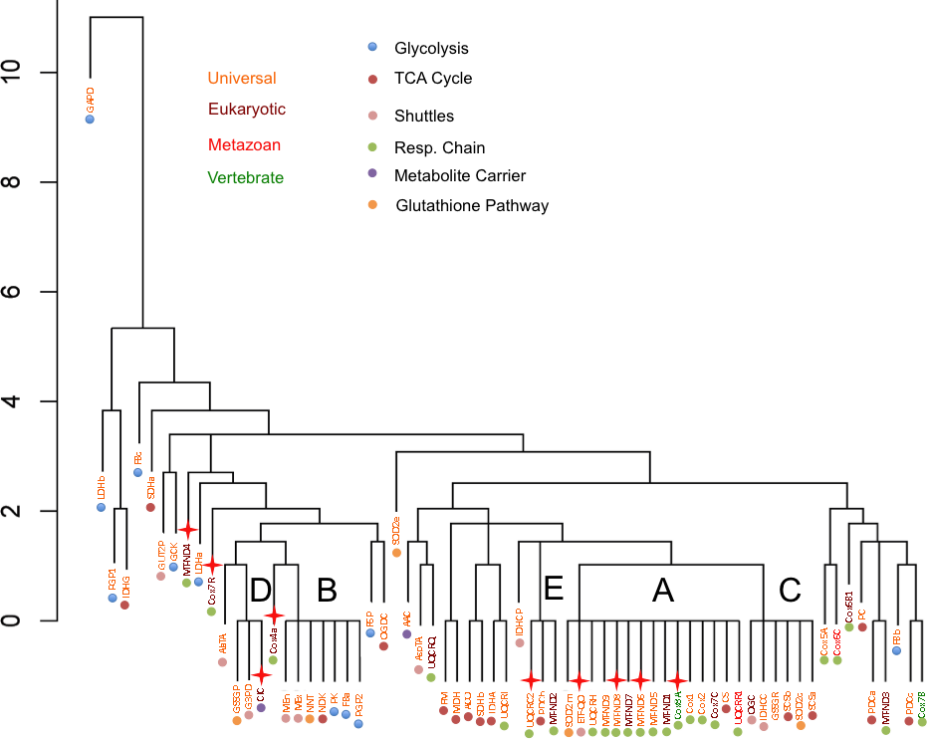
**Similarity of gene duplication histories in the GSIS system**. The cluster diagram is constructed from Euclidian distances between vectors of gene duplication history as described in the Materials and Methods. Gene names are coloured according to their phylogenetic classification (red = Metazoan, orange = Universal, brown = Eukaryotic, green = Vertebrate). Coloured dots represent pathway membership as shown in the key. Red stars indicate genes inferred to have undergone at least one episode of positive selection, according to the Selectome database (see Table 2 for more detail).

**Figure 5:**
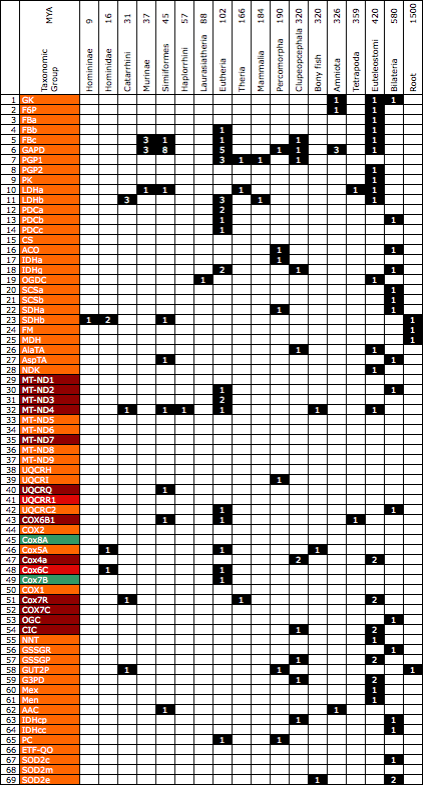
**Matrix of inferred gene duplications in the 69 GSIS genes**. Events are taken from the Ensembl gene tree displays and binned according to the taxonomic and date information given there. Bins with one or more inferred duplication are shaded, and numbers of inferred duplications at a given taxonomic level are given. These values were included in the calculation of the cluster diagram in Figure 4.

The cluster diagram in Figure 4 represents the similarities and differences in the duplication histories between the GSIS genes. Details of the individual duplication vectors are presented in Figure 5. Figure 4 shows five clusters of three or more gene histories (labelled A-E in the Figure). The largest of these (A in Figure 4) is dominated by respiratory chain genes, including the mitochondrially encoded Cox1 and Cox2, and represents a history without detectable duplication. The other groups are less functionally homogeneous. To investigate whether the hierarchy shows association of proteins within subpathways of the model or within phylogenetic groups, these subsets were compared with all remaining pairs to identify whether any showed a significant difference from expected values. Significant clustering was observed for respiratory chain proteins (P << 0.001; Mann-Whitney). Weaker clustering was observed for pyruvate/NADH shuttle and TCA cycle genes (P < 0.05) but these were not significant after Bonferroni correction. A significant anticlustering was observed for glycolysis genes compared to average gene pairs. This in part reflected a high amount of duplication observed for GAPD (glyceraldehyde 3-phosphate dehydrogenase), but was detectable even if GAPD was excluded from the analysis. No significant clustering was observed within phylogenetic categories but significant anticlustering was observed for the Universal class. This reflected the presence of GAPD in this class as when it was removed no significant anticlustering was observed.

After duplication, genes appear to continue to undergo purifying selection with their evolutionary rates and genomic persistence depending on their level of essentiality [21]. However, there are examples of positive selection acting on duplicated genes [22], presumably reflecting adaptive changes. We therefore investigated whether there was evidence of positive selection affecting GSIS genes. We identified seventeen possible episodes of positive selection, summarized in Table 2 and related to gene duplication history in Figure 5. Most of the inferred positive selection took place within respiratory chain genes and none within the glycolysis, TCA cycle and glutathione pathways. One respiratory chain gene, Cox4a, showed five inferred episodes of positive selection and also underwent two inferred gene duplication events which coincided approximately with one of these periods of positive selection, being placed at the root of the Euteleostomi c. 420 MYA. A second respiratory chain Cox gene, Cox7R, also showed coincident duplication and positive selection, in this case at the root of the Catarrhini 31 MYA (Table 2). To test the relationship between purifying selection and gene duplication we carried out rank correlation analysis of divergence rate versus number of duplications. This showed a significant positive correlation (r = 0.339; P = 0.004; 2-tailed) indicating that in this system as elsewhere genes that are more conserved tend to have undergone more duplication [23].

**Table 2:** Positive selection within GSIS

Protein	Sub-Pathway	Inferred node (approximate date)
MT-ND4	R	Percomorpha (190 MYA)
MT-ND6	R	Percomorpha
		Catarrhini (31MYA)
MT-ND8	R	Eutheria (102 MYA)
		Tetrapoda (359 MYA)
UQCRC2	R	Theria (166 MYA)
		Tetrapoda
		Clupeocephala (320 MYA)
Cox8A	R	Eutheria
Cox4A	R	Theria
		Murinae (37 MYA)
		Clupeocephala*
		Euteleostomi (420 MYA)†
Cox7R	R	Catarrhini†
CIC	M	Eutheria
ETF-QO	N	Percomorpha

* Two episodes inferred; † Coincides with a duplication

### Disease Association

24 of the genes analysed here are associated with Mendelian diseases in the OMIM database (Table 3). We found no relationship between pathway membership, phylogenetic category and disease involvement in the ANOVA analysis. We did however identify a deficit of genes that had undergone positive selection in the disease class (P = 0.01, Chi squared, 1 DOF).

**Table 3:** GSIS genes with disease associations

Gene	OMIM Code	Disease
GCK	125851	MODY, type II,
	125853	Insulin resistance, susceptibility to,
	602485	Hyperinsulinemic hypoglycemia, familial, 3
	606176	Diabetes, permanent neonatal
FBa	229600	Fructose intolerance
FBc	611881	Glycogen storage disease XII
PGP2	261670	Glycogen storage disease X
PK	102900	Adenosine triphosphate, elevated, of erythrocytes
	266200	Pyruvate kinase deficiency
PDCa	308930	Leigh syndrome, X-linked
	312170	Pyruvate dehydrogenase deficiency
PDCb	312170	Pyruvate dehydrogenase deficiency
OGDC	203740	Alpha-ketoglutarate dehydrogenase deficiency
SCSa	245400	Lactic acidosis, fatal infantile
SDHa	115310	Paraganglioma, familial chromaffin, 4
	171300	Pheochromocytoma, modifier of
	606864	Paraganglioma and gastric stromal sarcoma
	612359	Cowden-like syndrome
SDHb	252011	Mitochondrial respiratory chain complex II deficiency
	256000	Leigh syndrome, due to Cox deficiency
FM	150800	Multiple cutaneous and uterine leiomyomata
	605839	Leiomyomatosis and renal cell cancer
	606812	Fumarase deficiency
MT-ND1	252010	Mitochondrial complex I deficiency
MT-ND2	256000	Leigh syndrome, due to Cox deficiency
UQCRQ	124000	Mitochondrial complex III deficiency
Cox6B1	220110	Mitochondrial complex IV deficiency
Cox2	220110	Mitochondrial complex IV deficiency
Cox1	220110	Mitochondrial complex IV deficiency
GUT2P	125853	Insulin resistance, susceptibility to
IDHcc	137800	Glioblastoma, susceptibility to
PC	266150	Pyruvate carboxylase deficiency
ETF-QO	231680	Glutaricaciduria, type IIC
SOD2c	105400	Amyotrophic lateral sclerosis, susceptibility to
SOD2 m	612634	Microvascular complications of diabetes, susceptibility to

## Discussion

This study reveals a highly significant relationship between pathway membership and human-mouse divergence in the GSIS system. The analysis indicated two cross-pathway clusters of divergence - a higher conservation cluster spanning glycolysis, the TCA cycle plus metabolite transport and the components of the NADH shuttle and pyruvate cycle, and a lower conservation cluster comprising respiratory chain proteins and components of the glutathione pathway. All the genes considered, with the exceptions of the mitochondrially encoded Cox1 and Cox2 in Complex IV of the respiratory chain, are encoded by the nuclear genome, so a genome effect did not play a role in this result. It should be noted that, as we have considered protein similarity in these analyses, they cannot be used to directly infer differences of evolutionary rate at the DNA level as they also include the influence of selection on protein sequences.

The results are consistent with those of Vinogradov [13], who showed that the strongest predictor of the human-mouse divergence of a given protein was the evolutionary rate of coexpressed genes or members of the same pathway. In the context of the metabolic processes studied here, the two coevolving sets of proteins (or coalitions [13]) we have observed correspond to the core biochemistry of the system and the membrane-based ATP synthesis machinery, respectively. Previous work comparing 12 Drosophila genomes has also suggested that core metabolic enzymes tend to evolve slowly [24]. The similar rates of evolution in the glutathione pathway and the ATP synthesis machinery may reflect coevolution of these pathways. This would be explicable as the role of the glutathione pathway is to detoxify peroxide radicals produced by oxidative phosphorylation.

We also observed a second, weaker coevolutionary pattern than that of gene divergence within pathways. This was of evolutionary duplication within at least some of the pathways. Gene duplication is a classic mechanism of gene diversification which can be followed by functional diversification processes such as subfunctionalisation, neofunctionalisation and microfunctionalisation [25-27]. Thus, common patterns of preserved duplication during evolution may indicate responses to common evolutionary pressures. The cluster diagram (Figure 4) shows five clusters of size n > 3 with identical duplication histories. The largest of these, with 15 members (labelled A in Figure 4), predominantly contains respiratory chain proteins from Complexes I and IV and includes Cox1 and Cox2. The other two large groups B and C (with seven and six members each, respectively) show less homogeneous compositions. Group A contains genes which show no identifiable gene duplications as far back as the root of their respective gene family tree. This coincides with the observation that oxidative phosphorylation genes tend to show an under-representation of gene duplication [12]. The second largest group, with 7 members, (labelled B in Figure 4) corresponds to genes which underwent a duplication at or around the origin of the Euteleostomi but have not undergone a duplication since. Finally, the 6-member group (labelled C in Figure 4) represents another group showing little duplication, this time since around the origin of the Bilateria (c. 580 MYA). 40% of the GSIS proteins therefore show one of three common patterns of evolutionary duplication history involving either no detectable duplication or a single duplication deep in the evolutionary past. Beyond the presence of numerous respiratory chain proteins in the large cluster, visual inspection does not reveal significant clustering of pathway members or phylogenetic class members in this cluster diagram. Statistical analysis supported this conclusion but did however show significant anticlustering of glycolysis proteins, i.e. glycolysis proteins show a more diverse pattern of clustering history than average and this effect can be observed even if glyceraldehyde-3-phosphate dehydrogenase (GAPD) is excluded.

GAPD showed very high frequencies of gene duplication throughout its recent evolutionary history, leading to it being an outlier in Figure 4. Indeed, GAPD has been identified with an exceedingly high number of processed pseudogenes in vertebrates and possibly it is this which is reflected in its inferred duplication history [28]; certainly Ensembl identified 75 duplicates of GAPD in rat and 16 in mouse, consistent with the over 300 pseudogenes identified in these species [28]. The reasons for this high level of retrotransposition are not known, but may reflect the wider non-glycolytic role of GAPD or some as yet unidentified sequence feature of the GAPD cDNA sequence [28]. It has been suggested that non-pseudogene paralogs have been retained because of a role for GAPD in the bundling of microtubules and other cellular functions as well as functional diversification into testis- and muscle-specific forms [11].

Gene duplication is usually considered to be followed by a period of relaxed selection followed by the continuation of a regime of purifying selection [29]. The co-occurrence of gene duplication with episodes of positive selection would therefore be of interest in interpreting the functional evolution of a gene. We observed two such apparent co-occurrences: in Cox4A and Cox7R - both components of Complex IV of the respiratory chain. Cox4A is a transmembrane protein which binds ATP and mediates respiratory control by inhibiting cytochrome c oxidase activity at high intramitochondrial ATP concentrations [30]. Cox4A showed both duplication and positive selection at the base of the Euteleostomi c. 420 MYA but also additional positive selection, in the absence of gene duplication, on three separate occasions: in the fish lineage at the base of the Clupeocephala c. 320 MYA and in the land vertebrates at the origin of Theria (c. 166 MYA) and murinae (c. 37 MYA). This rich history of gene duplication suggests an important adaptive role for this gene. Cox7R, about which less is known but is also likely to be a regulatory subunit [30], underwent three duplications including one near to the origin of the Catarrhini (c. 31 MYA) which was also accompanied by an episode of positive selection.

These observations present us with two opposing views of the evolution of GSIS proteins: high conservation of core biochemical pathways with higher divergence of the respiratory chain, but also restricted duplication of respiratory chain genes compared to core biochemistry proteins, especially glycolytic enzymes which show the highest conservation. A correlation analysis showed a significant negative correlation between evolutionary divergence and numbers of gene duplications, although this only accounted for 11% of variance.

It has been suggested that "older" genes tend to evolve more slowly than "younger" ones [31] and there is evidence that newly-born duplicates evolve more rapidly than unduplicated genes [29,32]. Vinogradov [13] also identified gene age as an important correlate of divergence rate. In this analysis, we use phylogenetic category as a measure of gene age. Taken as a whole, the GSIS network is enriched in Universal genes that is, genes with detectable homologues in bacteria, compared to the whole metabolic complement of the human genome. This is consistent with the ancient nature of the biochemical pathways making up the system and with the slow evolution of the genes they contain. The glycolysis pathway, which is most conserved at the sequence level in human-mouse comparisons is, along with the glutathione pathway, also the richest in Universal genes. The respiratory chain, which is the most rapidly evolving set, has a much higher content of "younger" genes. Therefore, a close relationship appears to exist between gene age and evolutionary rate, with older genes evolving more slowly, as suggested elsewhere [31]. However, as ancient but rapidly evolving genes may be difficult or impossible to detect by BLAST-based methods [33], the phylogenetic categorization of genes used here may be strongly influenced by the rates of evolution of individual genes, which would explain the association of Universal genes with low human-mouse divergence. An additional possible confounding effect is inhomogeneity in evolutionary rate within proteins. Proteins which contain highly conserved cores interspersed with or flanked by highly variable sequences might show lower average conservation than proteins accepting substitutions evenly throughout their length but remain detectable by BLAST because of their conserved core. This should not affect alignment-based measures of sequence similarity where the distribution of conserved and non-conserved sites is immaterial but could affect these BLAST-based methods and should be investigated further.

Unlike Vinogradov [13], we did not find any significant relationship in ANOVA between phylogenetic category and either human-mouse divergence (P = 0.46) or number of gene duplications (P = 0.83) after accounting for pathway effects. This could reflect the smaller number of categories used in this analysis compared to the 12 used by Vinogradov [13] or the smaller number of pathways studied. Also, Vinogradov's categorization is based on Clusters of Orthologous Groups (COGs; [34]), which, although they are BLAST-based, make use of protein rather than DNA BLASTs. We conclude that we cannot exclude influences of gene age on evolutionary rate although it does not provide a significant signal in this analysis.

It has been suggested that disease genes tend to be "ancient" [35]. Of the 24 disease genes in the GSIS system 20 are Universal, but the proportion of Universal genes is not disproportionately high compared to the entire set of GSIS proteins (P = 0.68, Chi-square). Similarly, although disease genes in the GSIS system are on average more conserved than other genes (95.1% human-mouse similarity vs 93.1%), this difference is not statistically significant (P = 0.07; t-test). Thus, while we cannot exclude the hypothesis that disease genes tend to be old, we cannot find strong support for it in our data set. One property that was observed in this set of disease genes was a deficit of genes with a history of positive selection. This may be consistent with the suggestion that disease genes tend to be conserved as, by implication, they should also not have undergone accelerated evolution.

Although the main feature of Figure 3 is the pathway-dependent divergence of the GSIS proteins, it also identifies four clear outliers from three of the pathways: NDK and PDCc from the TCA cycle, IDHcp from the pyruvate cycle and SOD2e from the glutathione pathway. Three and perhaps all of these lie at the points of interaction between subpathways of the GSIS network (see Additional File 1 and Figure 2). NDK links to the output of the GSIS system, ATP production. It is activated by increasing levels of ADP, which in turn up-regulates the TCA cycle. PDCc is found at a clear intersection between glycolysis and the TCA cycle and is involved in the production of Acetyl-CoA, regulated by NAD^+ ^and CoA. The IDHcp intersection is less clear, but is one step in linking the TCA cycle to glycolysis, and appears to be involved in regulating the pyruvate-malate equilibrium in the cytosol. SOD2e is at a key step in the detoxification of reactive oxygen species generated by the respiratory chain and creates the intersection with the glutathione pathway by converting O_2_^- ^to H_2_O_2_.

Taken together, the picture we obtain from these analyses is that the GSIS system consists of two evolutionary coalitions, each evolving in a concerted manner. The soluble enzymes carrying out core metabolic processes, particularly glycolysis and the TCA cycle, evolve slowly at the sequence level but undergo a relatively high rate of gene duplication compared to the respiratory chain and glutathione pathway proteins, which evolve more rapidly but undergo relatively little gene duplication. The glycolysis pathway, although highly conserved at the sequence level, displays a wider range of duplication histories than other pathways. Steinke et al [11] analysed the patterns of gene duplication within the glycolysis pathway in some detail and concluded that some of the pattern of duplication seen in this pathway could be explained by diversification of tissues and the evolution of tissue-specific patterns of expression following on from two suggested gene duplication events (three in fish). However tissue-specific expression could not explain the complex pattern of gene duplication and retention/loss in its entirety. The high frequency of fixed duplications in the glycolysis pathway may reflect the fact that it is the only cytoplasmic pathway in the system. It may therefore need to adapt to a wider range of changes in other, interlinked pathways than, for example, the TCA cycle, which may be relatively protected from such pressures. In particular, glycolysis acts as the source of ATP in anaerobic respiration and so may adapt to the external availability of oxygen. It also appears to be used to drive DNA replication and cell division as it is less damaging to DNA as it does not produce reactive oxygen species [36].

Although our sample of positive selection may be biased, most positive selection episodes appear to have taken place in respiratory chain genes. The reason for the more rapid evolution of the respiratory chain genes is unknown, but it is worth noting that most of the proteins here are nuclearly encoded proteins involved in the regulation of the function of the respiratory chain [30]. It may therefore be that their rapid evolution reflects the requirement for fast adaptation of regulation of the respiratory chain in response to organismal pressures or to variations in the availability of oxygen. Some proteins in the network show particularly high rates of evolution and these tend to lie at the interfaces between recognizable pathways. These proteins may act as "adapters", facilitating the interactions between different pathways which may themselves evolve as units without coevolution between pathways. Adapters may then need to evolve rapidly to accommodate the divergence between the linked pathways.

Most of the functions of the component pathways of the GSIS network seem likely to have been conserved during evolution. Despite this the system has accommodated changes through sequence evolution and gene duplication. While it will require more extensive functional analysis to characterise the pressures driving the evolution of this system, it appears that these pressures have been accommodated in different ways by different parts of the system, as detailed above.

## Conclusions

The GSIS system consists of two evolutionary coalitions, each evolving in a concerted manner. The soluble enzymes carrying out core metabolic processes evolve slowly at the sequence level but undergo a relatively high rate of gene duplication compared to the respiratory chain and glutathione pathway proteins, which evolve more rapidly but undergo relatively little gene duplication. A small number of rapidly evolving proteins may represent evolutionary adapters, which mediate the different evolutionary trajectories of the different sub-pathways.

The comparative analysis of the GSIS network provides key insights into the evolution of the involved proteins that might be used in future research within a functional context, by simulating the function of the entire network in health and disease [7]. Elements of the system undergoing positive selection, rapid evolution more broadly, or gene duplication should be investigated for functional differences between humans and model organisms.

## Methods

### Data Collection

The analysis is based on the components of a mathematical model of the core biochemistry of mouse glucose-stimulated insulin secretion published previously [7], consisting of 77 enzymes. Of the 55 protein identifiers (IDs) listed in that model, 50 were included and their human orthologues retrieved. The five remaining mouse proteins not considered here are: pyruvate carrier (Q4A5HQ1), isocitrate dehydrogenase NAD+ beta (O43837), phosphate carrier (Q00325), aspartate/glutamate carrier (Q59GB0) and ATP/ADP carrier (P12236). This set was then augmented by an additional 19 human proteins, NADH:Ubiquinone oxidoreductase (9 components), ubiquinol:cytochrome c oxidoreductase (4 components), manganese-dependent superoxide dismutase 2 (3 components), nicotinamide nucleotide transhydrogenase (Q13423), glutathione peroxidase (P07203) and ATP/ADP Carrier 2 (P05141). The resulting 69 mouse-human orthologue pairs corresponding to components of the system were unambiguously identified using the Ensembl database (V52) [37]. As many of these are members of gene families, they were curated manually to ensure that the correct gene was being analyzed. Orthologous pairs of sequences were extracted from Swiss-Prot [38] based on unique identifiers (IDs) obtained from Ensembl. The set of 69 proteins analysed is summarised in Table 1. Sequence pairs from human and mouse were aligned using EMBL-EBI Align using the Needleman-Wunsch global alignment algorithm [39] and the BLOSUM62 transition matrix [40]. Pairwise sequence similarity values were then determined and analysed.

### Phylogenetic Assignment

Phylogenetic profiles [14] of human protein sequences involved in GSIS were estimated using BLAST searches [41] against 243 fully sequenced genomes from the COGENT database [42,43], including 197 Bacteria, 22 Archaea and 24 Eukaryota species, using an E-value cut-off of 10 (12,083 hits, of which 11,925 hits, or 98.7% of the total, had an E-value < 1). The classification process was non-hierarchical: proteins with hits to more than five prokaryote species are classified as universal [U]; the remaining proteins with at least a single hit to non-metazoan eukaryotes are classified as eukaryote-specific [E]; proteins with at least a single hit to non-mammalian metazoa are classified as metazoan-specific [M]; and, finally, proteins recognizing only other vertebrate proteins are classified as vertebrate-specific [V]. All proteins were classified into one of the above taxonomy classes using the last update of this analysis in COGENT. Profiles were also used to identify proteins with common ancestors in other genomes. These protein clusters were further analysed with reference to the Pfam database [44].

### Duplication History and Divergence Time Estimates

Gene trees obtained from Ensembl were screened visually for duplication events and used to construct duplication vectors for each gene. These vectors consisted of the number of duplications falling at nodes labelled with particular times and taxon names in Ensembl (e.g. Clupeocephala, 320 MYA). Vectors were only extended to the point at which a duplication created a new gene family, to avoid confusion with selective forces acting on proteins with different functions, and non-vertebrate duplications were excluded. Vectors were subjected to hierarchical clustering using the R *dist *and *hclust *functions and Euclidian distances [45]. Euclidian distances between subpathway members and between proteins classified into particular phylogenetic categories were compared with distances for all other pairwise comparisons to identify groups differing significantly from average in their degree of clustering, using the Mann-Whitney test.

### Selection Forces and Disease Association

Lineages showing evidence of positive selection were identified from the Selectome database [46] and confirmed using the Codeml program in the Paml (Phylogenetic Analysis by Maximum Likelihood) package [47]. Disease associations of GSIS genes were determined by reference to the OMIM (Online Mendelian Inheritance in Man) database [48]. Statistical analysis was carried out using the R environment [45].

## Authors' contributions

CA and JMH designed the research and outlined the manuscript. CA, MS, SF, OE and LH performed the computational analysis. CA, MS, LH and JMH interpreted the data. ST and CAO provided feedback on the results and contributed in drafting the manuscript. CA and JMH wrote the paper. All authors read and approved the final manuscript.

## Supplementary Material

Additional file 1 **Overview of the GSIS network**. Reactions are represented as arrows (which may be uni- or bidirectional) and labelled in green v1 to v53 according to [7] which also provides a key to abbreviations. Metabolites are labelled in black. Enzymes catalysing reactions are labelled in brown. Click here for file
