# Supplementary figures and images for: Protein coalitions in a core mammalian biochemical network linked by rapidly evolving proteins
Source: BMC Evol Biol. 2011 May 25;11:142. doi: 10.1186/1471-2148-11-142 (PMC3112093; doi:10.1186/1471-2148-11-142)

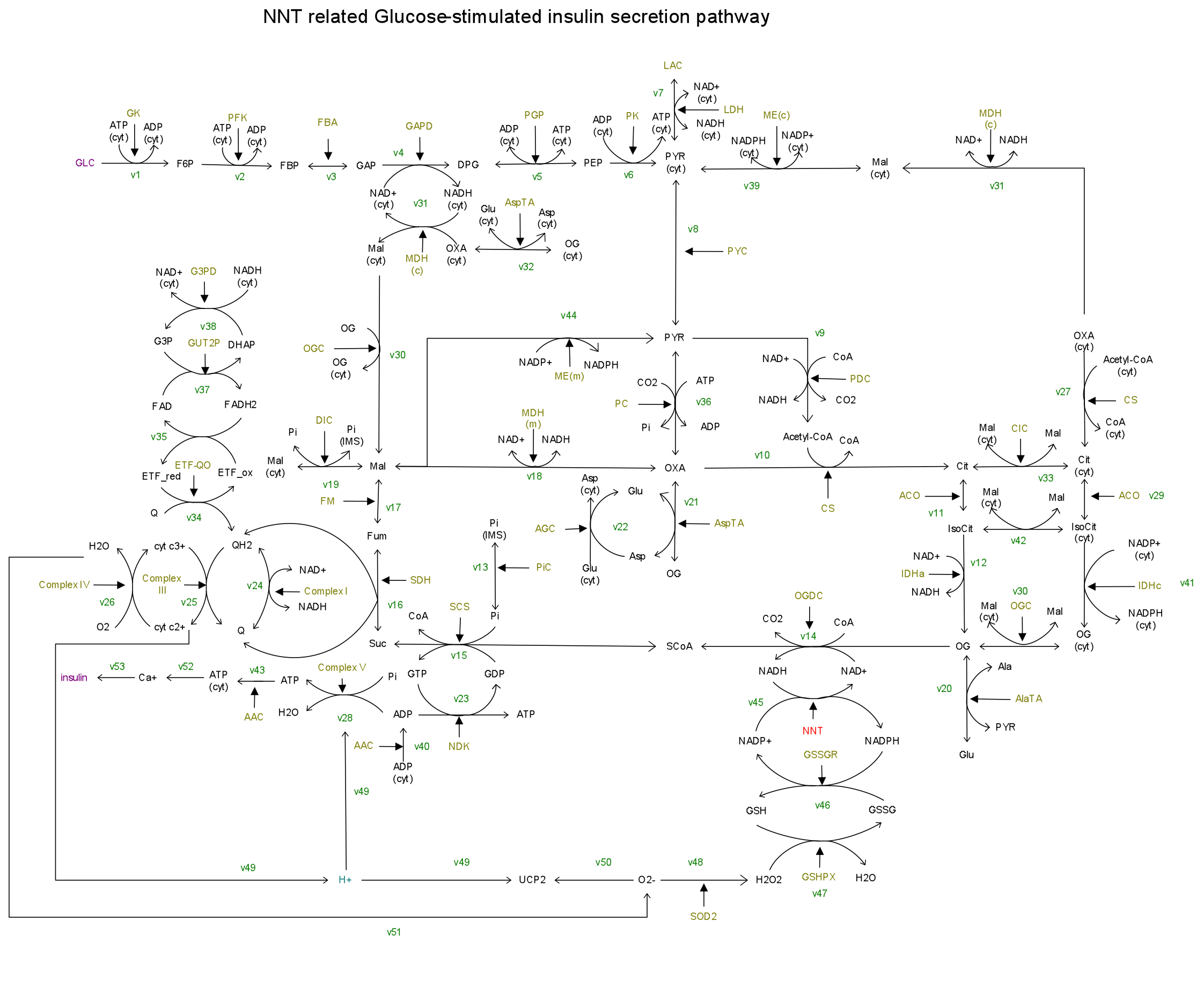

Supplement: Additional file 1 — Overview of the GSIS network. Reactions are represented as arrows (which may be uni- or bidirectional) and labelled in green v1 to v53 according to [7] which also provides a key to abbreviations. Metabolites are labelled in black. Enzymes catalysing reactions are labelled in brown. [file 1471-2148-11-142-S1.TIFF]
